# Supplementary material for: Arsenic exposure is associated with elevated sweat chloride concentration and airflow obstruction among adults in Bangladesh: A cross-sectional study
Source: PLoS One. 2025 May 7;20(5):e0311711. doi: 10.1371/journal.pone.0311711 (PMC12057939; doi:10.1371/journal.pone.0311711)
Supplement: S1 Table — (DOCX) [file pone.0311711.s001.docx]

**Supplementary Table 1.** Comparison of study population characteristics among those included in the PFT analysis compared to those for whom PFTs were not available, *n* (%) or mean ± standard deviation (SD).

| Variables | Participants with PFTs (*n* = 166) | Participants without PFTs ^†^ (*n* = 134) | *P*-value ^‡^ |
| --- | --- | --- | --- |
| Age, yrs | 51.3 ± 9.9 | 51.7 ± 11.6 | 0.70 |
| Sex |  |  |  |
| Male | 78 (47.0) | 89 (66.4) | <0.001 |
| Female | 88 (53.0) | 45 (33.6) |  |
| BMI | 22.7 ± 3.8 | 23.0 ± 3.5 | 0.36 |
| Education |  |  |  |
| Able to write | 71 (42.8) | 41 (30.6) | 0.02 |
| Primary education | 45 (27.1) | 33 (24.6) |  |
| Middle school or above | 50 (30.1) | 60 (44.8) |  |
| Smoking status |  |  |  |
| Current | 31 (18.7) | 26 (19.4) | 0.99 |
| Former | 20 (12.1) | 16 (11.9) |  |
| Never | 115 (69.3) | 92 (68.7) |  |

^†^ Subjects who either did not complete PFTs or did not have ATS acceptable PFTs

^‡^ *P-v*alue for comparing the differences in each variable between included (*n*=166) versus excluded (*n*=134), given by the Chi-square (χ^2^) test for categorical variables and *t-*test for continuous variables.
