# Supplementary material for: Arsenic exposure is associated with elevated sweat chloride concentration and airflow obstruction among adults in Bangladesh: A cross-sectional study
Source: PLoS One. 2025 May 7;20(5):e0311711. doi: 10.1371/journal.pone.0311711 (PMC12057939; doi:10.1371/journal.pone.0311711)
Supplement: S2 Table — (DOCX) [file pone.0311711.s002.docx]

**Supplementary Table 2.** Relationship between sweat chloride and age, sex, education and smoking status, *n* (%) or median [IQR].

| Variables | Overall  (*n =* 269) | Sweat chloride  (<60 mmol/L)  (*n* = 200) | Sweat chloride  (≥ 60 mmol/L)  (*n* = 69) | *P*-value^†^ |
| --- | --- | --- | --- | --- |
| Age, yrs | 52.0 [15] | 51.0 [17] | 55.0 [11] | 0.210 |
| Sex |  |  |  | <0.001 |
| Male | 147 (54.6) | 97 (48.5) | 50 (72.5) |  |
| Female | 122 (45.4) | 103 (51.5) | 19 (27.5) |  |
| Education |  |  |  | 0.203 |
| Able to write | 105 (39.0) | 81 (40.5) | 24 (34.8) |  |
| Primary education | 68 (25.3) | 45 (22.5) | 23 (33.3) |  |
| Middle school or above | 96 (35.7) | 74 (37.0) | 22 (31.9) |  |
| Smoking Status |  |  |  |  |
| Current | 49 (18.2) | 33 (16.5) | 16 (23.2) | <0.001 |
| Former | 32 (11.9) | 16 (8.0) | 16 (23.2) |  |
| Never | 188 (69.9) | 151 (75.5) | 37 (53.6) |  |

^†^ *P-v*alue for comparing the differences in each variable between sweat chloride
(<60 mmol/L) versus sweat chloride (≥ 60 mmol/L), given by the Chi-square (χ^2^) test for categorical variables and *t-*test for continuous variables.
