# Supplementary material for: Arsenic exposure is associated with elevated sweat chloride concentration and airflow obstruction among adults in Bangladesh: A cross-sectional study
Source: PLoS One. 2025 May 7;20(5):e0311711. doi: 10.1371/journal.pone.0311711 (PMC12057939; doi:10.1371/journal.pone.0311711)
Supplement: S3 Table — (DOCX) [file pone.0311711.s003.docx]

**Supplementary Table 3.** *CFTR* sequencing results

| Participant No. | Age, y | Sex | Drinking water arsenic concentration (µg/L) | Nail arsenic concentration (µg/g) | Sweat chloride concentration (mmol/L) | Reportable *CFTR* variant | *CFTR* Deletion or Duplication |
| --- | --- | --- | --- | --- | --- | --- | --- |
| 1 | 32 | M | 45.0 | 8.2 | 66.0 | None | None |
| 2 | 34 | M | 130.0 | 5.9 | 72.0 | None | None |
| 3 | 35 | M | 171.0 | 4.1 | 61.0 | None | None |
| 4 | 38 | M | 632.0 | 17.2 | 86.0 | None | None |
| 5 | 40 | M | 0.5 | 0.7 | 88.0 | None | None |
| 6 | 42 | F | 305.0 | 2.8 | 68.0 | None | None |
| 7 | 45 | F | 1.0 | 4.5 | 75.0 | None | None |
| 8 | 45 | F | 98.0 | 11.6 | 90.0 | None | None |
| 9 | 46 | M | 28.0 | 4.3 | 68.0 | None | None |
| 10 | 47 | M | 63.0 | 4.8 | 98.0 | None | None |
| 11 | 47 | F | 221.0 | 9.8 | 64.0 | None | None |
| 12 | 48 | M | 124.0 | 11.0 | 62.0 | None | None |
| 13 | 49 | M | 0.0 | 4.7 | 62.0 | None | None |
| 14 | 50 | M | 66.0 | 9.0 | 75.0 | None | None |
| 15 | 50 | M | 200.0 | 7.1 | 72.0 | Heterozygous 5T variant within the poly-thymidine tract in intron 9 of the CFTR gene (polyT mutation) (polyT-TG:12TG-7T polyT-TG:13TG-5T) | None |
| 16 | 50 | M | 22.0 | 1.5 | 66.0 | None | None |
| 17 | 50 | F | 127.0 | 16.8 | 64.0 | None | None |
| 18 | 50 | F | 42.0 | 13.5 | 70.0 | None | None |
| 19 | 50 | F | 353.0 | 3.7 | 70.0 | None | None |
| 20 | 51 | F | 364.0 | 8.4 | 75.0 | None | None |
| 21 | 51 | F | 23.2 | 1.9 | 70.0 | None | None |
| 22 | 51 | F | 38.0 | 21.2 | 82.0 | None | None |
| 23 | 52 | F | 123.0 | 3.6 | 87.0 | None | None |
| 24 | 54 | F | 1077.0 | 27.0 | 84.0 | p.R1070Q mutation (c.3209G>A p.R1070Q [HET] [311.267.0:q6495]) | None |
| 25 | 55 | M | 0.0 | 2.0 | 60.0 | Heterozygous 5T variant within the poly-thymidine tract in intron 9 of the CFTR gene (polyT mutation) (polyT-TG:11TG-9T polyT-TG:13TG-5T) | None |
| 26 | 55 | M | 275.0 | 13.8 | 65.0 | p.F508del mutation (c.1521_1523delCTT p.F508del [HET] [242.244.0:q9406] c.1408G>A p.V470M [HET] [264.227.0:q5293]) | Deletion |
| 27 | 55 | F | 81.0 | 3.2 | 81.0 | None | None |
| 28 | 55 | F | 14.0 | 0.5 | 70.0 | None | None |
| 29 | 55 | M | 395.0 | 10.5 | 75.0 | None | None |
| 30 | 56 | F | 95.0 | 2.6 | 84.0 | None | None |
| 31 | 56 | M | 55.0 | 1.5 | 92.0 | Heterozygous 5T variant within the poly-thymidine tract in intron 9 of the CFTR gene (polyT mutation) (polyT-TG:12TG-5T polyT-TG:11TG-7T) | None |
| 32 | 56 | M | 162.4 | 15.1 | 84.0 | None | None |
| 33 | 56 | M | 203.0 | 7.2 | 90.0 | None | None |
| 34 | 57 | F | 69.0 | 3.3 | 70.0 | None | None |
| 35 | 58 | M | 45.0 | 4.1 | 78.0 | None | None |
| 36 | 58 | F | 489.0 | 6.0 | 81.0 | None | None |
| 37 | 58 | M | 452.0 | 6.4 | 60.0 | None | None |
| 38 | 58 | M | 5.0 | 0.8 | 75.0 | None | None |
| 39 | 60 | M | 69.0 | 4.1 | 60.0 | None | None |
| 40 | 65 | F | 5.0 | 12.9 | 65.0 | None | None |
| 41 | 65 | M | 653.0 | 31.5 | 67.0 | None | None |
| 42 | 67 | M | 101.0 | 4.2 | 70.0 | Heterozygous 5T variant within the poly-thymidine tract in intron 9 of the CFTR gene (polyT mutation) (polyT-TG:13TG-5T polyT-TG:11TG-7T) | None |
| 43 | 74 | M | 29 | 9.2 | 78.0 | None | None |
| 44 | 75 | M | 167 | 18.9 | 72.0 | None | None |
| 45 | 75 | M | 4 | 2.3 | 63.0 | None | None |
