# Supplementary material for: Arsenic exposure is associated with elevated sweat chloride concentration and airflow obstruction among adults in Bangladesh: A cross-sectional study
Source: PLoS One. 2025 May 7;20(5):e0311711. doi: 10.1371/journal.pone.0311711 (PMC12057939; doi:10.1371/journal.pone.0311711)
Supplement: S4 Table — (DOCX) [file pone.0311711.s004.docx]

**Supplementary Table 4.** Adjusted effect estimates (β and 95% CIs) ^†^ from multivariable regression models for lung function and odds ratios (ORs and 95% CIs) ^†^ from logistic regression models for airflow obstruction associated with an IQR increase in toenail arsenic among never smokers (*n* = 115)

| **Toenail arsenic** | **FEV_1_** | | **FVC** | | **FEV_1_/FVC** | | **Airflow obstruction** | | |
| --- | --- | --- | --- | --- | --- | --- | --- | --- | --- |
|  | β (95% CI) | *P*-value | β (95% CI) | *P*-value | β (95% CI) | *P*-value | Cases/  Non-cases | OR (95% CI) | *P*-value |
| 2001-2003 | -0.03 (-0.09, 0.03) | 0.31 | -0.02 (-0.08, 0.04) | 0.50 | -0.01 (-0.02, 0.003) | 0.14 | 10/105 | 1.97 (1.13, 3.45) | 0.02 |
| 2018-2021 | -0.05 (-0.13, 0.03) | 0.23 | -0.04 (-0.12, 0.05) | 0.36 | -0.01 (-0.02, 0.01) | 0.21 | 10/105 | 2.40 (1.02, 5.65) | 0.05 |
| Long-term^†^ | -0.06 (-0.15, 0.03) | 0.17 | -0.04 (-0.13, 0.04) | 0.32 | -0.01 (-0.03, 0.002) | 0.08 | 10/105 | 3.97 (1.44, 10.90) | 0.008 |

^†^ Models adjusted for age, sex (male and female), height, and education (able to write, primary education, and above middle school). Note: IQR, interquartile range.

^‡^ Average of toenail arsenic concentrations in 2001-2003 and 2018-2021.
