# Supplementary material for: Arsenic exposure is associated with elevated sweat chloride concentration and airflow obstruction among adults in Bangladesh: A cross-sectional study
Source: PLoS One. 2025 May 7;20(5):e0311711. doi: 10.1371/journal.pone.0311711 (PMC12057939; doi:10.1371/journal.pone.0311711)
Supplement: S5 Table — (DOCX) [file pone.0311711.s005.docx]

| Variables | Participants with PFTs (*n* = 166) | | | |
| --- | --- | --- | --- | --- |
|  | All  (*n* = 166) | Airflow Obstruction  (*n* = 33) | No Airflow Obstruction  (*n* = 133) | *P*-value^†^ |
| Age, yrs | 51.3 ± 9.87 | 54.5 ± 9.7 | 50.5 ± 9.8 | 0.032 |
| Sex |  |  |  |  |
| Female | 88 (53.0) | 6 (18.2) | 82 (61.7) | 0.255 |
| Male | 78 (47.0) | 27 (81.8) | 51 (38.5) | reference |
| Height | 22.65 ± 3.83 | 20.9 ± 2.9 | 23.1 ± 3.9 | 0.397 |
| Education |  |  |  |  |
| Middle school or above | 50 (30.1) | 11 (33.3) | 39 (29.3) | 0.275 |
| Primary education | 45 (27.1) | 9 (27.3) | 36 (27.1) | 0.865 |
| Able to write | 71 (42.8) | 13 (39.4) | 58 (43.6) | reference |
| Smoking status |  |  |  |  |
| Current smoker | 31 (18.7) | 14 (42.4) | 17 (12.8) | 0.087 |
| Former | 20 (12.1) | 9 (27.3) | 11 (8.3) | 0.727 |
| Never | 115 (69.3) | 10 (30.3) | 105 (78.9) | reference |

**Supplementary Table 5:** Association between airflow obstruction and age, sex, height, education and smoking status.

^†^ *P*-value for comparing the difference between airflow obstruction versus no airflow obstruction, given by logistic regression analysis after adjusting for all covariates and toenail arsenic concentrations in 2001-2003 and 2018-2021.
