# Supplementary material for: Arsenic exposure is associated with elevated sweat chloride concentration and airflow obstruction among adults in Bangladesh: A cross-sectional study
Source: PLoS One. 2025 May 7;20(5):e0311711. doi: 10.1371/journal.pone.0311711 (PMC12057939; doi:10.1371/journal.pone.0311711)
Supplement: S1 Fig — (DOCX) [file pone.0311711.s006.docx]

**Supplementary Figure 1.**  Distribution of long-term drinking water and toenail arsenic concentrations among participants with normal/intermediate (<60 mmol/L) and abnormal (≥60 mmol/L) sweat chloride concentration.

**
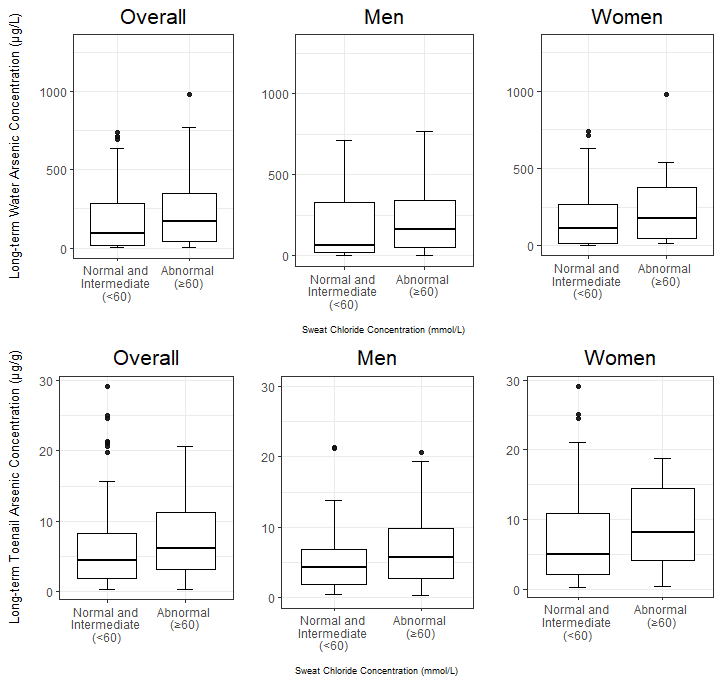
**
