# Supplementary material for: Arsenic exposure is associated with elevated sweat chloride concentration and airflow obstruction among adults in Bangladesh: A cross-sectional study
Source: PLoS One. 2025 May 7;20(5):e0311711. doi: 10.1371/journal.pone.0311711 (PMC12057939; doi:10.1371/journal.pone.0311711)

**Supplementary Figure 2.** Odds ratios (ORs) and 95% confidence intervals (CIs) for abnormal sweat chloride at different cutoff levels associated with concurrent toenail arsenic concentration. All models are adjusted for sex and smoking status.


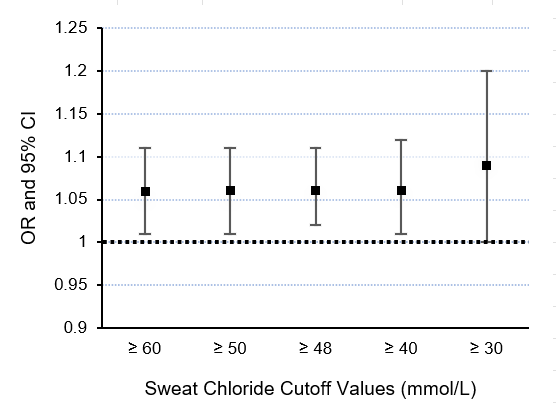

Supplement: S2 Fig — All models are adjusted for sex and smoking status. (DOCX) [file pone.0311711.s007.docx]
