# Supplementary material for: Arsenic exposure is associated with elevated sweat chloride concentration and airflow obstruction among adults in Bangladesh: A cross-sectional study
Source: PLoS One. 2025 May 7;20(5):e0311711. doi: 10.1371/journal.pone.0311711 (PMC12057939; doi:10.1371/journal.pone.0311711)

**Supplementary Figure 4.** Mediation analysis of the long-term arsenic-lung function by sweat chloride. A, Mediation for FEV_1_; B, Mediation for FVC; C, Mediation for FEV_1_/FVC; D, Mediation for airway obstruction. Models were adjusted for age, sex, height, smoking status, and education.


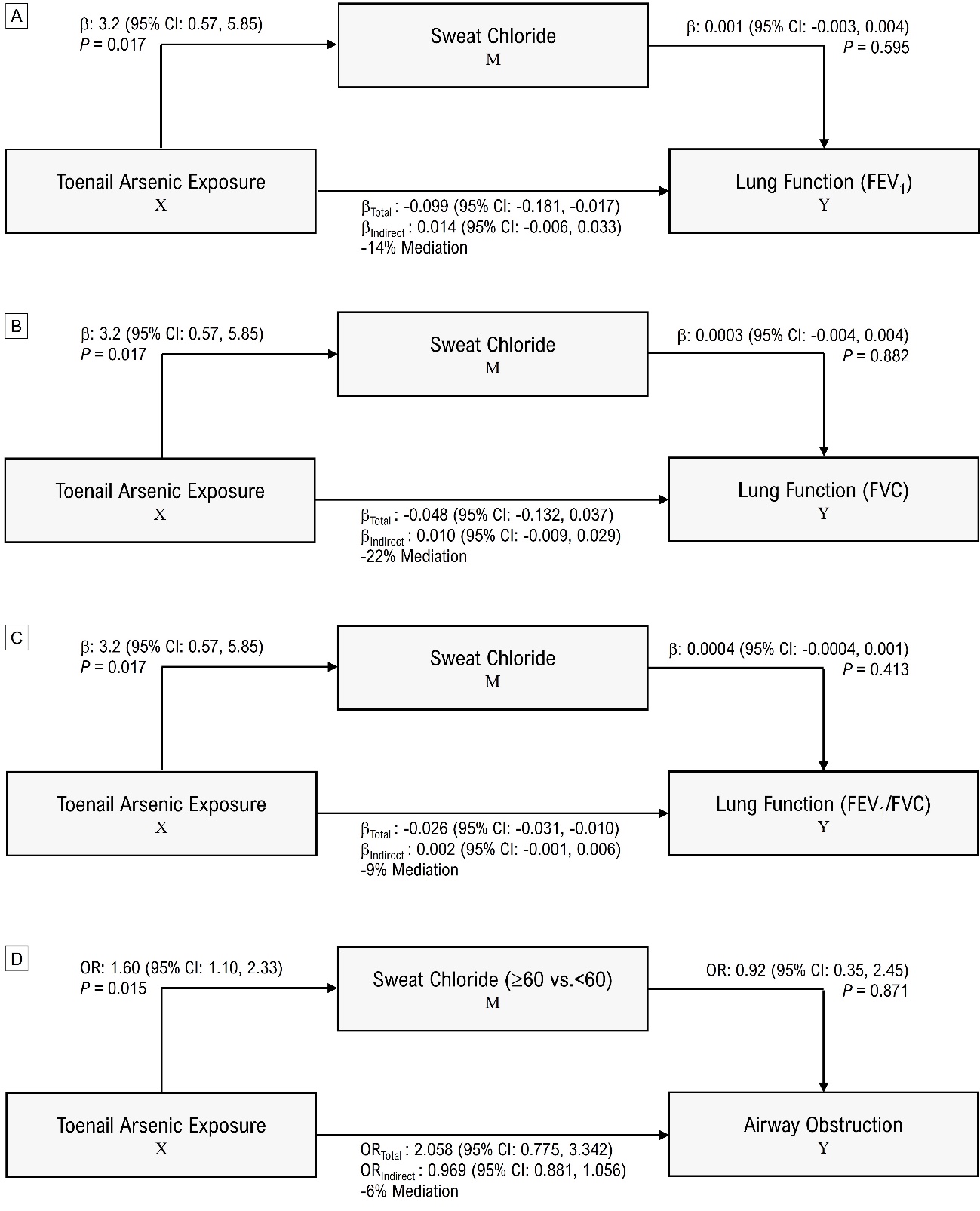

Supplement: S4 Fig — (A) Mediation for FEV1; (B) Mediation for FVC; (C) Mediation for FEV1/FVC; (D) Mediation for airway obstruction. Models were adjusted for age, sex, height, smoking status, and education. (DOCX) [file pone.0311711.s009.docx]
