# Supplementary material for: Arsenic exposure is associated with elevated sweat chloride concentration and airflow obstruction among adults in Bangladesh: A cross-sectional study
Source: PLoS One. 2025 May 7;20(5):e0311711. doi: 10.1371/journal.pone.0311711 (PMC12057939; doi:10.1371/journal.pone.0311711)
Supplement: Attachment — Submitted filename: response - arsenic exposure and sweat chloride.pdf [file pone.0311711.s010.pdf]

The manuscript titled "Arsenic exposure is associated with elevated sweat chloride concentration and airflow obstruction among adults in Bangladesh: a cross-sectional study" investigates the link between arsenic exposure, CFTR dysfunction (as measured by sweat chloride levels), and pulmonary function in Bangladeshi adults. The authors hypothesize that arsenic exposure impairs CFTR function, reflected in elevated sweat chloride concentrations, and that this dysfunction may mediate arsenic's impact on lung function. Through comprehensive measurements of arsenic levels in toenails and water, sweat chloride testing, pulmonary function testing, and CFTR genotyping, the study aims to establish sweat chloride as a biomarker for arsenic exposure and explore the mechanisms underlying arsenic-associated lung disease. This study is significant due to its focus on an underexplored potential biomarker (sweat chloride) for arsenic exposure and its implications for screening arsenic-related diseases in low-resource settings. However, the manuscript should only be considered after proper adjustments and corrections have been made including clarity in methodology, coherence in data interpretation, and justification of certain scientific claims. Below, I provide detailed feedback on areas for improvement, corrections, and suggestions for clarifications.

1) The abstract effectively summarizes the study but lacks clarity in certain statements. For example, the line "toenail arsenic concentration was associated with increased odds of airway obstruction" is significant, yet the mediating role of sweat chloride is dismissed briefly without adequate line of explanation. Consider expanding the abstract to succinctly state why sweat chloride concentration does not mediate lung obstruction and what alternative mechanisms may be involved.
2) The introduction frames the problem well, highlighting the global relevance of arsenic exposure. The authors draw a clear connection between arsenic-induced CFTR dysfunction and cystic fibrosis pathology, establishing a solid foundation for their hypothesis. But my concern is that the introduction states, "Recent studies in killfish demonstrate that arsenic induces CFTR ubiquitinylation and degradation." While the killfish model is relevant, its application to humans requires further justification. Are there other human studies that corroborate this? I would suggest a sentence to contextualize the translational relevance of the killfish findings to humans.
3) The claim, "These findings suggest that CFTR dysfunction is unlikely to be the primary mechanism of arsenic-associated lung disease," is valid but incomplete. The alternative

mechanisms (e.g., oxidative stress, CC16 reduction) mentioned later should be brought forward earlier in the discussion.

4) For the participant recruitment, the inclusion of participants from a 2001-2003 cohort raises concerns about representativeness. Did any external factors between 2003 and 2018-2022 potentially affect arsenic exposure levels? Clarify if this could bias results. Same for the CFTR sequence analysis; you stated that, "None of the DNA samples tested revealed homozygous pathogenic mutations in CFTR." However, the relevance of this analysis is unclear when heterozygous mutations are briefly mentioned but not discussed further. How do the authors interpret these findings in the context of CFTR dysfunction? Please clarify the rationale behind choosing the 2001-2003 cohort and address the potential influence of time on arsenic exposure levels. Also, elaborate on how heterozygous CFTR variants might contribute to the observed findings, or explicitly state why they were excluded from further discussion.
5) The finding, "airflow obstruction was present among 19.9% (n=33)," is significant but would benefit from more exploration. Were other factors (e.g., age, smoking, BMI) contributing to this result? Why were only 45 out of 69 participants with elevated sweat chloride included in CFTR sequencing? Does this partial analysis introduce bias?
6) It is unclear why the authors chose toenail arsenic concentration as the primary exposure metric over drinking water arsenic, especially when toenail arsenic correlates moderately with water arsenic ($r = 0.66$). Justify the prioritization of toenail arsenic as the primary measure in the text.
7) The authors state, "Sweat tests may be an effective, inexpensive, point-of-care screening test for arsenic exposure." While promising, this conclusion requires more evidence, particularly regarding the feasibility of implementing this in rural settings. The sentence, "diagnoses of cystic fibrosis made only by sweat tests... may be false positives," is intriguing but underexplored. Could these findings influence current diagnostic practices? Expand on the challenges of implementing sweat chloride testing in low-resource areas and its diagnostic implications for cystic fibrosis.
8) The manuscript occasionally assumes knowledge from the reader (e.g., the significance of certain heterozygous CFTR mutations). Simplify technical jargon where possible and provide additional context for less familiar concepts.
9) How do the authors account for potential confounders such as nutrition or comorbidities that may influence sweat chloride or lung function? Could the authors

clarify whether the sweat chloride measurements were repeated to ensure reliability? What steps were taken to ensure that toenail arsenic levels reflect long-term exposure rather than recent changes in arsenic intake?

10) The abstract mentions odds ratios (e.g., "OR: 1.97, 95% CI: 1.06, 3.67") but does not specify the subset analyzed (e.g., never-smokers, entire cohort). Including this detail would make it clearer. Specify whether results are based on the full cohort or sub-cohorts (e.g., never-smokers) for transparency.
11) The supplementary data supports the introduction's framing of arsenic as a major health hazard with plausible mechanisms for CFTR involvement. Revise the claim "Sweat chloride may be a biomarker for arsenic exposure" to emphasize that this is a novel hypothesis requiring further validation. Maybe say "Sweat chloride may serve as a novel biomarker for arsenic exposure, warranting further investigation in diverse populations."
12) The inclusion of heterozygous CFTR mutations raises questions about partial CFTR dysfunction. How did the authors interpret the presence of heterozygous variants in individuals with high sweat chloride levels?
13) There is a significant sex imbalance between participants with and without PFTs ($p < 0.001$). Could this skew the interpretation of lung function results? Address whether sex was a key confounder or if the analyses were stratified.
14) The analysis for airflow obstruction among never-smokers is strong but under-reported in the discussion.
15) Authors adjusted for several covariates, but potential environmental factors (e.g., air pollution, occupational exposure) are not mentioned. Address whether these factors were considered or discuss their potential as unmeasured confounders.
16) I have made some annotations and highlights on the manuscript provided below. These markings indicate sections that require proper rewriting, corrections, or the addition of further information to enhance clarity and ensure a better understanding for the reader. Please address these areas as suggested to improve the overall quality of the manuscript.

Title: Arsenic exposure is associated with elevated sweat chloride concentration and airflow obstruction among adults in Bangladesh: a cross sectional study

Mi-Sun S. Lee[1],* Crystal M. North[1,2],* Irada Choudhuri[3], Subrata K. Biswas[4], Abby F. Fleisch[5,6], Afifah Farooque[7], Diane Bao[7], Sakila Afroz[8], Sadia Mow[8], Nazmul Husain[8], Fuadul Islam[8], Md Golam Mostafa[8], Partha Pratim Biswas[9], David S. Ludwig[10], Subba R. Digumarthy,[11] Christopher Hug[12], Quazi Quamruzzaman[8], David C. Christiani[1,2], Maitreyi Mazumdar[1,7]

*Co-first author. Drs. Lee and North had equal contributions to this work.

[1]Department of Environmental Health, Harvard T.H. Chan School of Public Health, Boston, MA USA

[2]Division of Pulmonary and Critical Care Medicine, Massachusetts General Hospital, Boston, MA, USA

[3]Department of Medicine, University of Pittsburgh Medical Center, Pittsburgh, PA, USA

[4]Department of Molecular and Cell Biology, University of Connecticut, Storrs, CT, USA

[5]Center for Interdisciplinary Population Health Research, MaineHealth, Portland, ME USA

[6]Pediatric Endocrinology and Diabetes, Maine Medical Center, Portland, ME USA

[7] Department of Neurology, Boston Children’s Hospital, Boston, MA USA

[8]Dhaka Community Hospital Trust, Dhaka Bangladesh

[9]Department of Biochemistry, Bangabandhu Sheikh Mujib Medical University, Dhaka, Bangladesh

[10]New Balance Obesity Prevention Center, Boston Children's Hospital, Boston, MA USA

[11]Thoracic Imaging and Intervention Division, Massachusetts General Hospital, Boston, MA USA

[12]Consultant, Brookline, MA, USA

Funding Information:

We received funding from the National Institute of Environmental Health Sciences (R01 ES027825, R01 ES011622, P30 ES000002) and the National Heart, Lung, and Blood Institute (K23 HL154863).

Abstract

Arsenic is associated with lung disease and experimental models suggest that arsenic-induced degradation of the chloride channel CFTR (cystic fibrosis transmembrane conductance regulator) is a mechanism of arsenic toxicity. We examined associations between arsenic exposure, sweat chloride concentration (measure of CFTR function), and pulmonary function among 285 adults in Bangladesh. Participants with sweat chloride ≥ 60 mmol/L had higher arsenic exposures than those with sweat chloride < 60 mmol/L (water: median 77.5 µg/L versus 34.0 µg/L, $p = 0.025$; toenails: median 4.8 µg/g versus 3.7 µg/g, $p = 0.024$). In linear regression models, a one-unit µg/g increment in toenail arsenic was associated with a 0.59 mmol/L higher sweat chloride concentration, $p < 0.001$. We found that toenail arsenic concentration was associated with increased odds of airway obstruction (OR: 1.97, 95%: 1.06, 3.67, $p = 0.03$); however, sweat chloride concentration did not mediate this association. Our findings suggest that sweat chloride concentration may be a novel biomarker for arsenic exposure and also that arsenic likely acts on the lung through mechanisms other than CFTR dysfunction.

INTRODUCTION

Up to 220 million people in as many as 70 countries are exposed to elevated arsenic levels through contaminated groundwater.[1,2] Arsenic exposure has been associated with lung dysfunction,[3-8] respiratory symptoms,[9-11] bronchiectasis,[12] and increased tuberculosis-related[13] and respiratory-related mortality.[14] One proposed mechanism for arsenic's effects on the lung is impairment of mucociliary clearance.

The cystic fibrosis transmembrane conductance regulator (CFTR) is a cyclic adenosine monophosphate-regulated chloride channel in the apical membrane of airway epithelial cells. CFTR plays an essential role in mucociliary clearance by establishing an osmotic gradient across the airway epithelium that promotes fluid secretion.[15-17] Mutations in *CFTR*, the gene that encodes CFTR, cause cystic fibrosis, a rare autosomal recessive disease that affects the lungs and digestive system. Cystic fibrosis is also associated with a diminished immune response to some gram-negative bacteria; a similarly-diminished immune response is induced by arsenic exposure.[18,19]

Recent studies in killfish demonstrate that arsenic induces CFTR ubiquitinylation and degradation.[20,21] In human airway epithelial cell culture, arsenic induces an increase in multiubiquitinlyated CFTR, which results in CFTR degradation and reduced CFTR-mediated chloride secretion.[22] Together, these reports demonstrate that arsenic impairs CFTR function and support the hypothesis that there is a shared pathobiologic mechanism driving both cystic fibrosis and arsenic-induced lung disease.

In humans, CFTR function is directly measured by a sweat test, which is the gold standard diagnostic test for cystic fibrosis.[23] CFTR mediates chloride resorption from sweat; in the absence or impaired function of CFTR, sweat chloride concentration is

elevated. In a recent study of 100 adults in Bangladesh – a country with among the highest arsenic exposure in the world – water and toenail arsenic concentrations were higher among adults with increased sweat conductivity (a marker for elevated sweat chloride concentrations), and those with the highest sweat conductivity did not have *CFTR* mutations consistent with a genetic diagnosis of cystic fibrosis.[24] This finding in humans supports the hypothesis that arsenic induces CFTR degradation, as was seen in experimental models, and that CFTR degradation may be a mechanism of arsenic toxicity.

Although sweat conductivity is correlated with sweat chloride concentration, the latter remains the gold standard for identifying CFTR dysfunction.[25] Therefore, to evaluate the relationship between arsenic exposure, CFTR dysfunction, and lung function, we measured arsenic levels, sweat chloride concentrations, and lung function among adults in Bangladesh. Our study aimed to test the hypotheses that higher arsenic exposure is associated with CFTR dysfunction as assessed by sweat chloride concentrations, and that CFTR dysfunction mediates the relationship between arsenic exposure and lung dysfunction.

All protocols were reviewed and approved by the institutional review boards (IRBs) of Boston Children's Hospital (BCH) (Protocol number: IRB-P00024501), the Dhaka Community Hospital Trust (DCH), and the Bangabandhu Sheikh Mujib Medical University (BSMMU) (Protocol number: BSMMU/2019/3739). The Harvard T.H. Chan School of Public Health (HSPH) and Massachusetts General Hospital (MGH) relied on BCH IRB review. All participants provided written informed consent.

METHODS

Study participants

From 13/05/2018 through 15/11/2022, we enrolled 300 adults at Dhaka Community Hospital. These participants were recruited from a total of 1800 adults who had participated in studies of arsenic and skin lesions between 2001 and 2003 in Pabna, Bangladesh.[26] We paused recruitment and study visits for one week between 20/12/2020 and 27/12/2020 while renewal of IRB applications was under review. No participants were enrolled from BSMMU. BSMMU laboratories were used to evaluate de-identified samples. We began by creating a list of potential participants using random digit assignment and contacted each potential participant in order on the list until we had reached our target enrollment. All participants from the 2001-2003 study were initially considered eligible for recruitment. Exclusion criteria included evidence of lung cancer or active pulmonary tuberculosis on chest radiography.

Arsenic exposure measurements

We collected drinking water samples from each participant's home. Water arsenic concentrations were measured in the Environmental Engineering Laboratory at the Bangladesh University of Engineering and Technology using graphite furnace atomic absorption spectrometry (GF-AAS).[27] Drinking water arsenic concentrations from the 2001-2003 study were measured using inductively coupled plasma mass spectrometry (ICP-MS) by the Environmental Laboratory Services (North Syracuse, NY, USA).[26]

We collected toenail clippings from all ten toes on a white sheet of paper, placed them in a small coin envelope (ULINE Model No. S-7798) and stored them at room

temperature. The Dartmouth Trace Element core facility measured arsenic in toenails using ICP-MS.[28] Arsenic concentrations in toenail clippings from the 2001-2003 study were measured at the Harvard T.H. Chan School of Public Health using similar ICP-MS protocols.[29]

Sweat chloride measurements

We performed sweat collection and sweat chloride testing according to established guidelines.[30] Briefly, we obtained sweat from the ventral surface of both forearms of each participant. After cleaning the skin with distilled water, we stimulated sweat secretion by iontophoresis (total applied current 1.5 mA, 50μA/cm^2) for five minutes using 0.5% pilocarpine gel disks (Pilogels®) and sweat was collected for 30 minutes in a Macroduct® Sweat Collection System (Wescor Biomedical Systems, Logan, UT). The laboratory measured sweat chloride concentrations within 48 hours at the Department of Biochemistry, Bangabandhu Sheikh Mujib Medical University (BSMMU) using a Dimension® RxL Max® Clinical Chemistry Analyzer (Siemens Healthcare Diagnostics Inc., Newark, DE) with QuikLYTE® Integrated Multisensor Technology (IMT). The LOD and coefficient of variation (CV) were 10 mmol/L and 1.1%, respectively. Samples with sweat volumes less than 15μL were considered insufficient for analysis.

DNA Isolation and *CFTR* Sequence Analysis

For participants with sweat chloride concentrations ≥ 60 mmol/L, we retrieved archived DNA samples that were obtained in the 2001-2003 study. DNA had been extracted from whole blood samples using the Puregene DNA Isolation kit (Gentra

Systems, Minneapolis, MN) and then stored at -80°C. We sent retrieved DNA samples to Ambry Genetics® (Ambry Test: CF; Aliso Viejo, CA) for *CFTR* sequence analysis and deletion/duplication analysis.[31]

Pulmonary Function Testing (PFT) and questionnaires

We measured lung function using EasyOne® Air handheld spirometers (Medical Technologies, Andover, MA). All tested participants had abstained from smoking for at least 45 minutes prior to testing and completed testing in the seated position after loosening any tight-fitting clothing. Measured parameters included forced expiratory volume in one second (FEV_1) and forced vital capacity (FVC), and all participants repeated PFTs after receiving four puffs of salbutamol (Beximco Pharmaceuticals, Bangladesh) and waiting for 10 minutes. Daily quality control procedures included multi-flow calibration confirmation using a 3-litre syringe (ndd Medical Technologies, Andover, MA). We stopped performing PFTs in March 2020, midway through participant enrollment, for the safety of participants and study staff because of the COVID-19 pandemic.

Two study investigators manually reviewed all PFTs for ATS acceptability and reproducibility standards.[32,33] We included PFTs in the analytic cohort if there were at least two acceptable maneuvers and both the FEV_1 and FVC from the two best trials were within 200mL of one another. We defined airflow obstruction as post-bronchodilator $FEV_1/FVC < 0.7$.

We used structured questionnaires to collect information on age, education, occupation, smoking history, and medications. We characterized respiratory symptoms

using the American Thoracic Society's Division of Lung Disease questionnaire (ATS-DLD 1978).[34] We measured height and weight at the study visit.

Chest Radiographs

All participants underwent posteroanterior and lateral chest radiographs performed by a trained radiology technician at DCH. All radiographs were evaluated by a DCH staff radiologist who was unaware of arsenic exposure, sweat chloride, or PFT results. The staff radiologist evaluated the radiographs for the presence of parenchymal, bony, or cardiac abnormalities. For participants who were diagnosed with airflow obstruction, a senior radiologist provided additional imaging review using a structured data collection form.

Statistical Analysis

We first examined all variables using descriptive statistics and assessed potential differences in characteristics between groups using *t*-tests for continuous variables and Chi-square tests for categorical variables. We computed Spearman correlation coefficients (*r*) between water and toenail arsenic concentrations. For samples below the limit of detection (LOD), we estimated water arsenic concentrations as LOD/2. We used the average of the toenail arsenic concentrations from the 2001-2003 and 2018-2022 visits to estimate long-term arsenic exposure. Our primary exposure of interest was toenail arsenic concentration. We modeled toenail arsenic using untransformed values and interquartile ranges (IQR).

We used multivariable linear regression to characterize the relationship between arsenic and sweat chloride. We evaluated for potential confounding effects by age, sex, smoking status (current, former, and never), and education (able to write, primary education, middle school or above) by placing each variable in the models individually and assessing for change in the regression coefficient for the arsenic measure.

We also used logistic regression to estimate the relationship between toenail arsenic concentration and the odds of having a sweat chloride concentration $\geq$ 60 mmol/L. We chose this threshold to be consistent with diagnostic criteria for cystic fibrosis. We considered toenail arsenic concentration our primary measure of arsenic exposure. In sensitivity analyses, we tested the above-described relationships using lower sweat chloride thresholds ($\geq$ 50 mmol/L, $\geq$ 40 mmol/L, and $\geq$ 30 mmol/L), as these lower thresholds are sometimes used to identify those who need additional evaluation for cystic fibrosis.[11]

We then characterized the relationship between toenail arsenic and lung function using multivariable linear regression (FEV_1, FVC, FEV_1/FVC) or logistic regression (FEV_1/FVC < 0.7), adjusting for age, sex, smoking status, education, and height (in centimeters). Lastly, we employed mediation analyses to estimate the degree to which sweat chloride mediates the relationship between long-term arsenic exposure and lung function, adjusting for age, sex, smoking status, education, and height.[35] Results are presented as regression coefficients (β with 95% confidence interval [95% CI]) for continuous outcomes and odds ratio (OR with 95% CI) for dichotomous outcomes per IQR increment in toenail arsenic concentrations. In sensitivity analyses, we restricted

analyses to never smokers to rule out the potential masking effects of smoking on the relationship between arsenic and lung function.

We performed statistical analyses using SAS (version 9.4; SAS Institute, Inc.), R (version 4.2.2; R Development Core Team), and RStudio (version 2022.12.0+353) software.

RESULTS

Study population

Of the 300 enrolled participants, 31 participants were excluded due to missing toenail clippings (n=10) or insufficient sweat volume (n=21), for a total of 269 (90%) participants. Participant characteristics are shown in **Table 1**. Of these 269 participants, 166 (62%) completed PFTs that met ATS acceptability and reproducibility criteria prior to the COVID19-related shutdown. The 166 participants who underwent PFTs were more frequently women (53% vs 33.6% p<0.001) and generally less educated (p=0.02) than the 134 participants for whom PFTs were not performed; otherwise there were no significant differences between these groups (**Supplementary Table 1**).

Arsenic concentrations

The distributions of drinking water and toenail arsenic concentrations are shown in **Table 2**. Overall, drinking water and toenail arsenic concentrations in 2018-2022 were lower than those observed in 2001-2003 (water median 41.0 μg/L in 2018-2021 compared with 71.2 μg/L in 2001-2003), reflecting the impact of arsenic awareness and reduction activities. We observed no significant sex differences in drinking water or toenail arsenic concentrations. All toenail arsenic concentrations were above the LOD of

0.002 μg/g. Toenail arsenic concentrations were moderately correlated with drinking water arsenic concentrations ($r = 0.66$, $p < 0.0001$).

Sweat chloride concentrations

Mean ± SD sweat chloride concentration was 49.1 ± 16.8 (median: 48.0 mmol/L, range: 18.0 to 98.0 mmol/L). A total of 69 (25.7%) participants had sweat chloride concentration ≥ 60 mmol/L. Sweat chloride concentrations were higher in men than women (51.6 ± 16.8 vs. 46.1 ± 16.4, p=0.007) and higher among smokers vs. never smokers (52.6 ± 16.5 vs. 47.6 ± 16.8). There were no significant associations between sweat chloride concentration, participant's age and education. (**Supplementary Table 2**).

CFTR Sequence Analysis

Of the 69 participants with sweat chloride ≥ 60 mmol/L, 45 (65.2%) underwent *CFTR* sequence analysis. None of the DNA samples tested revealed homozygous pathogenic mutations in *CFTR*. Six participants had heterozygous findings consistent with classification as cystic fibrosis carriers (**Supplementary Table 3**). These findings suggest that the elevated sweat chloride concentrations we observed were not caused by *CFTR* mutations that lead to absent or abnormal CFTR protein production.

Pulmonary function tests, respiratory symptoms, and chest imaging

No participant was found to have lung cancer, active pulmonary tuberculosis, cardiac or bony abnormalities on chest films. Of the 166 who underwent PFTs, mean ±

SD was 1.97 ± 0.54 L for FEV_1, 2.57 ± 0.67 for FVC, and 0.77 ± 0.09 for FEV_1/FVC (**Table 3**). Airflow obstruction was present among 19.9% (n=33), most of whom were men (27 of 33, 82%). Most of the 27 men with airflow obstruction (n=23, 85%) had a history of smoking, while none of the six women with airflow obstruction reported a history of smoking. A total of 55 participants (33.1%) endorsed respiratory symptoms, which included wheezing, shortness of breath or coughing. Respiratory symptoms were more prevalent among those with airflow obstruction; 57.6% of people with airflow obstruction reported respiratory symptoms compared to 27.1% of people without airflow obstruction. Among the 33 participants with airflow obstruction identified on PFTs, additional review of chest imaging showed evidence of bronchiectasis in 5 individuals.

Arsenic and sweat chloride

To evaluate sweat chloride's potential use as a biomarker, we present the most parsimonious models including modeling arsenic on a natural scale for ease of interpretation. Concurrent drinking water and toenail arsenic concentrations were higher among participants with sweat chloride ≥ 60 mmol/L compared to < 60 mmol/L (water: median 77.0 μg/L versus 34.0 μg/L, p = 0.025; toenails: median 4.8 μg/g versus 3.7 μg/g, p = 0.024) (**Figure 1**). A similar pattern was seen for long-term arsenic concentration (**Supplemental Figure 1).** In models adjusted for sex and smoking status, a one-unit μg/g increment in toenail arsenic concentration was associated with a 0.59 mmol/L higher sweat chloride concentration, p< 0.001, and a one-unit μg/g increment in toenail arsenic concentration was associated with greater odds of having a sweat chloride concentration ≥ 60 mmol/L (OR: 1.06, 95% CI, 1.01, 1.11). We found similar

findings at lower thresholds of sweat chloride. Models examining varying sweat chloride cutoffs are shown in **Supplemental Figure 2** and **Supplemental Figure 3**. In models fully adjusted for covariates, an IQR (6.6 μg/g) increment in long-term exposure to toenail arsenic was associated with a 3.2 mmol/L (95% CI: 0.57, 5.85, p = 0.017) higher sweat chloride concentration (**Supplementary Figure 4**).

Arsenic and pulmonary function tests

In adjusted models, an IQR increment in toenail arsenic concentration at each timepoint was associated with nonsignificant reductions in FEV_1, FVC, and FEV_1/FVC (**Table 4**). An IQR increment in toenail arsenic concentration from 2001-2003 was associated with greater odds of airflow obstruction (toenail arsenic 2001-2003 OR: 1.57, 95% CI: 1.03, 2.40, p = 0.03; long-term toenail arsenic (OR: 1.97, 95%: 1.06, 3.67, p = 0.03). Results were similar when we restricted the analytic cohort to never smokers (**Supplementary Table 4**).

Mediation analysis

Mediation analyses indicate that there is no evidence of sweat chloride as a mediator of the relationship between toenail arsenic concentrations and airflow obstruction (**Supplementary Figure 4**).

DISCUSSION

Our study demonstrates that 1) elevated sweat chloride concentration, the diagnostic hallmark of cystic fibrosis, is found among individuals exposed to arsenic through drinking water, 2) individuals with sweat chloride concentrations ≥ 60 mmol/L

did not have a genetic diagnosis of cystic fibrosis, 3) long-term arsenic exposure as estimated by toenail arsenic concentration is associated with airflow obstruction, and 4) sweat chloride concentration did not mediate the association between arsenic and airflow obstruction. These findings suggest that high sweat chloride may be a novel biomarker of arsenic exposure, but that CFTR dysfunction is unlikely to be the primary mechanism of arsenic-associated lung disease.

Our study has multiple implications for screening of arsenic-related disease in countries such as Bangladesh. Sweat tests may be an effective, inexpensive, point-of-care screening test for arsenic exposure. As we have demonstrated here and has been shown elsewhere,[24] it is possible to perform sweat tests in rural areas without expensive laboratory equipment. Another implication of our finding is that diagnoses of cystic fibrosis made only by sweat tests (i.e., without genetic testing, as is done in many low and middle income countries including Bangladesh[36]) may be false positives – that is, individuals' elevated sweat chloride concentrations may be a result of high arsenic exposure and not genetic disease.

Our findings showing associations between arsenic and airway obstruction are consistent with the published literature describing the relationship between arsenic exposure and lung dysfunction. Cross-sectional and cohort studies among childhood and adult populations in China, Mexico, and the United States consistently demonstrate relationships between arsenic exposure and restrictive patterns on lung function testing that are suggestive of restrictive lung disease.[37-40] Indeed, in a meta-analysis of nine studies totaling 4,699 participants in Bangladesh, Chile, India, Mexico, and Pakistan, higher arsenic exposure as measured by urine or drinking water concentrations was

associated with reduced FEV_1 and FVC, but not FEV_1/FVC.[41] In contrast, we found a relationship between arsenic and airflow obstruction (reduced FEV_1/FVC), and statistically insignificant reductions in FEV_1 and FVC. Relying upon spirometry to diagnose restrictive lung disease must be done with caution: total lung capacity (TLC) is the diagnostic gold standard to diagnose restrictive lung disease, and although FVC and TLC are related lung measurements, FVC-based algorithms unreliably identify restrictive lung disease.[42]

We did not find evidence that sweat chloride mediates the relationship between arsenic exposure and airflow obstruction, which suggests that there are mechanisms other than CFTR dysfunction that may explain arsenic's associations with lung health. For example, arsenic has been shown to induce direct lung tissue injury in tracheal epithelial cells and alveolar macrophages through the generation of reactive oxygen species and stress proteins.[43,44] Arsenic exposure is also associated with decreased serum CC16 levels,[45] which is a lung protective protein secreted by alveolar epithelial cells that modulates inflammatory lung injury. Lastly, arsenic exposure is associated with increased matrix metalloproteinase-9, one of a class of proteins that regulate airway inflammation and have been implicated in the pathogenesis of obstructive lung disease.[46-48]

In a similar manner, better understanding of the mechanisms of arsenic toxicity may benefit individuals with genetically-determined cystic fibrosis. Not all the individuals in our study who were exposed to high concentrations of arsenic had elevated sweat chloride concentrations or lung dysfunction, suggesting that other factors may be associated with preserved epithelial chloride transport. These factors might reveal

protective mechanisms that could be exploited to develop therapies for genetically-determined cystic fibrosis. The recent work in killfish and cell culture described above[20-22] suggests such mechanisms might include suppressed cellular response of ubiquitinylation.

The strengths of our study include our direct measurements of individual-level long-term arsenic exposure using toenail clippings, sweat chloride concentrations, lung function, and – among those with elevated sweat chloride concentrations – genotyping to exclude pathogenic *CFTR* variants. Our study also has limitations. Firstly, as with all cross-sectional studies, because sweat chloride concentrations were measured at the same time that water and toenail samples were collected, we cannot confirm that arsenic exposure preceded their elevated sweat chloride concentrations. However, the prevalence of *CFTR* mutations in South Asian populations is low.[49] This, in combination with the bench literature supporting CFTR dysfunction as a plausible mechanism through which arsenic exposure causes high sweat chloride concentrations, we believe our observations likely represent a true association with arsenic exposure. We also obtained PFTs in only a subset of the total study population because the COVID-19 pandemic interrupted PFT testing. However, there were no substantive differences in the participants who completed PFTs as compared to those who did not, so we do not anticipate that this biased our results. Finally, we were unable to characterize other pulmonary features of cystic fibrosis such as bronchiectasis, a finding that would be most evident on CT imaging as compared to plain films, or non-pulmonary features of cystic fibrosis such as pancreatic dysfunction. Future work directly measuring CFTR function in affected tissues including lung epithelium and neutrophils may be informative.

CONCLUSIONS

Environmental arsenic exposure is associated with impaired CFTR function, as demonstrated by elevated sweat chloride concentrations, and airway obstruction. Potential applications of our study include future work aimed at development of a point-of-care sweat chloride test as a biomarker of arsenic exposure.

REFERENCES

1. Ravenscroft P, Brammer H, Richards KS. *Arsenic pollution : a global synthesis*. RGS-IBG book series. Wiley-Blackwell; 2009:xxvii, 588 p.

2. Podgorski J, Berg M. Global threat of arsenic in groundwater. *Science*. May 22 2020;368(6493):845-850. doi:10.1126/science.aba1510

3. Chattopadhyay BP, Mukherjee AK, Gangopadhyay PK, Alam J, Roychowdhury A. Respiratory effect related to exposure of different concentrations of arsenic in drinking water in West Bengal, India. *Journal of environmental science & engineering*. Apr 2010;52(2):147-54.

4. Dauphine DC, Ferreccio C, Guntur S, et al. Lung function in adults following in utero and childhood exposure to arsenic in drinking water: preliminary findings. Research Support, N.I.H., Extramural

Research Support, Non-U.S. Gov't. *Int Arch Occup Environ Health*. Aug 2011;84(6):591-600. doi:10.1007/s00420-010-0591-6

5. Nafees AA, Kazi A, Fatmi Z, Irfan M, Ali A, Kayama F. Lung function decrement with arsenic exposure to drinking groundwater along River Indus: a comparative cross-sectional study. Comparative Study. *Environ Geochem Health*. Apr 2011;33(2):203-16. doi:10.1007/s10653-010-9333-7

6. Parvez F, Chen Y, Brandt-Rauf PW, et al. Nonmalignant respiratory effects of chronic arsenic exposure from drinking water among never-smokers in Bangladesh. Research Support, N.I.H., Extramural. *Environmental health perspectives*. Feb 2008;116(2):190-5. doi:10.1289/ehp.9507

7. De BK, Majumdar D, Sen S, Guru S, Kundu S. Pulmonary involvement in chronic arsenic poisoning from drinking contaminated ground-water. *J Assoc Physicians India*. May 2004;52:395-400.

8. Parvez F, Chen Y, Yunus M, et al. Arsenic exposure and impaired lung function. Findings from a large population-based prospective cohort study. Research Support, N.I.H., Extramural. *Am J Respir Crit Care Med*. Oct 1 2013;188(7):813-9. doi:10.1164/rccm.201212-2282OC

9. Mazumder DN, Haque R, Ghosh N, et al. Arsenic in drinking water and the prevalence of respiratory effects in West Bengal, India. Research Support, Non-U.S. Gov't

Research Support, U.S. Gov't, Non-P.H.S.

Research Support, U.S. Gov't, P.H.S. *International journal of epidemiology*. Dec 2000;29(6):1047-52.

10. Parvez F, Chen Y, Brandt-Rauf PW, et al. A prospective study of respiratory symptoms associated with chronic arsenic exposure in Bangladesh: findings from the Health Effects of Arsenic Longitudinal Study (HEALS). Research Support, N.I.H., Extramural. *Thorax*. Jun 2010;65(6):528-33. doi:10.1136/thx.2009.119347

11. Milton AH, Hasan Z, Rahman A, Rahman M. Non-cancer effects of chronic arsenicosis in Bangladesh: preliminary results. *Journal of environmental science and health Part A, Toxic/hazardous substances & environmental engineering*. Jan 2003;38(1):301-5.

12. Mazumder DN, Steinmaus C, Bhattacharya P, et al. Bronchiectasis in persons with skin lesions resulting from arsenic in drinking water. Research Support, N.I.H., Extramural

Research Support, U.S. Gov't, P.H.S. *Epidemiology*. Nov 2005;16(6):760-5.

13. Smith AH, Marshall G, Yuan Y, Liaw J, Ferreccio C, Steinmaus C. Evidence from Chile that arsenic in drinking water may increase mortality from pulmonary tuberculosis. Research Support, N.I.H., Extramural. *American journal of epidemiology*. Feb 15 2011;173(4):414-20. doi:10.1093/aje/kwq383

14. Argos M, Parvez F, Rahman M, et al. Arsenic and lung disease mortality in Bangladeshi adults. Research Support, N.I.H., Extramural. *Epidemiology*. Jul 2014;25(4):536-43. doi:10.1097/EDE.0000000000000106

15. Riordan JR, Rommens JM, Kerem B, et al. Identification of the cystic fibrosis gene: cloning and characterization of complementary DNA. *Science*. Sep 8 1989;245(4922):1066-73. doi:10.1126/science.2475911

16. Rommens JM, Iannuzzi MC, Kerem B, et al. Identification of the cystic fibrosis gene: chromosome walking and jumping. *Science*. Sep 8 1989;245(4922):1059-65.

17. Tarran R, Button B, Boucher RC. Regulation of normal and cystic fibrosis airway surface liquid volume by phasic shear stress. *Annu Rev Physiol*. 2006;68:543-61. doi:10.1146/annurev.physiol.68.072304.112754

18. Kozul CD, Hampton TH, Davey JC, et al. Chronic exposure to arsenic in the drinking water alters the expression of immune response genes in mouse lung. *Environ Health Perspect*. Jul 2009;117(7):1108-15. doi:10.1289/ehp.0800199

19. Kozul CD, Ely KH, Enelow RI, Hamilton JW. Low-dose arsenic compromises the immune response to influenza A infection in vivo. *Environ Health Perspect*. Sep 2009;117(9):1441-7. doi:10.1289/ehp.0900911

20. Shaw JR, Bomberger JM, VanderHeide J, et al. Arsenic inhibits SGK1 activation of CFTR Cl- channels in the gill of killifish, Fundulus heteroclitus. *Aquat Toxicol*. Jun 10 2010;98(2):157-64. doi:10.1016/j.aquatox.2010.02.001

21. Stanton CR, Thibodeau R, Lankowski A, Shaw JR, Hamilton JW, Stanton BA. Arsenic inhibits CFTR-mediated chloride secretion by killifish (Fundulus heteroclitus) opercular membrane. *Cell Physiol Biochem*. 2006;17(5-6):269-78. doi:10.1159/000094139

22. Bomberger JM, Coutermarsh BA, Barnaby RL, Stanton BA. Arsenic promotes ubiquitinylation and lysosomal degradation of cystic fibrosis transmembrane conductance regulator (CFTR) chloride channels in human airway epithelial cells. *J Biol Chem*. May 18 2012;287(21):17130-17139. doi:10.1074/jbc.M111.338855

23. Gibson LE, Cooke RE. A test for concentration of electrolytes in sweat in cystic fibrosis of the pancreas utilizing pilocarpine by iontophoresis. *Pediatrics*. Mar 1959;23(3):545-9.

24. Mazumdar M, Christiani DC, Biswas SK, Ibne-Hasan OS, Kapur K, Hug C. Elevated sweat chloride levels due to arsenic toxicity. *N Engl J Med*. Feb 5 2015;372(6):582-4. doi:10.1056/NEJMc1413312

25. Farrell PM, White TB, Ren CL, et al. Diagnosis of Cystic Fibrosis: Consensus Guidelines from the Cystic Fibrosis Foundation. *J Pediatr*. Feb 2017;181S:S4-S15 e1. doi:10.1016/j.jpeds.2016.09.064

26. Breton CV, Houseman EA, Kile ML, et al. Gender-specific protective effect of hemoglobin on arsenic-induced skin lesions. *Cancer Epidemiol Biomarkers Prev*. May 2006;15(5):902-7. doi:10.1158/1055-9965.EPI-05-0859

27. Frisbie SH, Mitchell EJ, Yusuf AZ, et al. The development and use of an innovative laboratory method for measuring arsenic in drinking water from western Bangladesh. *Environ Health Perspect*. Sep 2005;113(9):1196-204. doi:10.1289/ehp.7974

28. O'Brien KM, White AJ, Sandler DP, Jackson BP, Karagas MR, Weinberg CR. Do Post-breast Cancer Diagnosis Toenail Trace Element Concentrations Reflect Prediagnostic Concentrations? *Epidemiology*. Jan 2019;30(1):112-119. doi:10.1097/EDE.0000000000000927

29. Chen KL, Amarasiriwardena CJ, Christiani DC. Determination of total arsenic concentrations in nails by inductively coupled plasma mass spectrometry. *Biol Trace Elem Res*. Feb 1999;67(2):109-25. doi:10.1007/BF02784067

30. LeGrys VA, Applequist R, Briscoe DR, et al. Sweat testing: sample collection and quantitative chloride analysis: approved guideline. *Clin Lab Stand Ins*. 2009;20(Suppl 27):C34-A2.

31. Deignan JL, Astbury C, Cutting GR, et al. CFTR variant testing: a technical standard of the American College of Medical Genetics and Genomics (ACMG). *Genet Med*. Aug 2020;22(8):1288-1295. doi:10.1038/s41436-020-0822-5

32. Miller MR, Hankinson J, Brusasco V, et al. Standardisation of spirometry. *Eur Respir J*. Aug 2005;26(2):319-38. doi:10.1183/09031936.05.00034805

33. Pellegrino R, Viegi G, Brusasco V, et al. Interpretative strategies for lung function tests. *Eur Respir J*. Nov 2005;26(5):948-68. doi:10.1183/09031936.05.00035205

34. Ferris BG. Epidemiology Standardization Project (American Thoracic Society). *Am Rev Respir Dis*. Dec 1978;118(6 Pt 2):1-120.

35. VanderWeele TJ. A unification of mediation and interaction: a 4-way decomposition. *Epidemiology*. Sep 2014;25(5):749-61. doi:10.1097/EDE.0000000000000121

36. Sarkar PK, Akand N, Tahura S, et al. Antimicrobial sensitivity pattern of children with cystic fibrosis in Bangladesh: a lesson from a specialized Sishu (Children) Hospital. *Egyptian Pediatric Association Gazette*. 2022;70(33)doi:https://doi.org/10.1189/s43054-022-00127

37. Zhou M, Wang X, Yang S, et al. Cross-sectional and longitudinal associations between urinary arsenic and lung function among urban Chinese adults. *Sci Total Environ*. Oct 20 2022;844:157028. doi:10.1016/j.scitotenv.2022.157028

38. Recio-Vega R, Gonzalez-Cortes T, Olivas-Calderon E, Lantz RC, Gandolfi AJ, Gonzalez-De Alba C. In utero and early childhood exposure to arsenic decreases lung function in children. *J Appl Toxicol*. Apr 2015;35(4):358-66. doi:10.1002/jat.3023

39. Signes-Pastor AJ, Notario-Barandiaran L, Guill M, et al. Prenatal exposure to metal mixtures and lung function in children from the New Hampshire birth cohort study. *Environ Res*. Dec 1 2023;238(Pt 2):117234. doi:10.1016/j.envres.2023.117234

40. Powers M, Sanchez TR, Grau-Perez M, et al. Low-moderate arsenic exposure and respiratory in American Indian communities in the Strong Heart Study. *Environ Health*. Nov 28 2019;18(1):104. doi:10.1186/s12940-019-0539-6

41. Sanchez TR, Powers M, Perzanowski M, George CM, Graziano JH, Navas-Acien A. A Meta-analysis of Arsenic Exposure and Lung Function: Is There Evidence of Restrictive or Obstructive Lung Disease? *Curr Environ Health Rep*. Jun 2018;5(2):244-254. doi:10.1007/s40572-018-0192-1

42. Swanney MP, Beckert LE, Frampton CM, Wallace LA, Jensen RL, Crapo RO. Validity of the American Thoracic Society and other spirometric algorithms using FVC and forced expiratory volume at 6 s for predicting a reduced total lung capacity. *Chest*. Dec 2004;126(6):1861-6. doi:10.1378/chest.126.6.1861

43. Cohen DS, Palmer E, Welch WJ, Sheppard D. The response of guinea pig airway epithelial cells and alveolar macrophages to environmental stress. *Am J Respir Cell Mol Biol*. Aug 1991;5(2):133-43. doi:10.1165/ajrcmb/5.2.133

44. Han SG, Castranova V, Vallyathan V. Heat shock protein 70 as an indicator of early lung injury caused by exposure to arsenic. *Mol Cell Biochem*. Sep 2005;277(1-2):153-64. doi:10.1007/s11010-005-5874-y

45. Lantz RC, Chau B, Sarihan P, Witten ML, Pivniouk VI, Chen GJ. In utero and postnatal exposure to arsenic alters pulmonary structure and function. *Toxicol Appl Pharmacol*. Feb 15 2009;235(1):105-13. doi:10.1016/j.taap.2008.11.012

46. Christopoulou ME, Papakonstantinou E, Stolz D. Matrix Metalloproteinases in Chronic Obstructive Pulmonary Disease. *Int J Mol Sci*. Feb 14 2023;24(4)doi:10.3390/ijms24043786

47. Wells JM, Parker MM, Oster RA, et al. Elevated circulating MMP-9 is linked to increased COPD exacerbation risk in SPIROMICS and COPDGene. *JCI Insight*. Nov 15 2018;3(22)doi:10.1172/jci.insight.123614

48. Atkinson JJ, Senior RM. Matrix metalloproteinase-9 in lung remodeling. *Am J Respir Cell Mol Biol*. Jan 2003;28(1):12-24. doi:10.1165/rcmb.2002-0166TR

49. Rafique H, Safdar A, Ghani MU, et al. Exploring the diversity of CFTR gene mutations in cystic fibrosis individuals of South Asia. *J Asthma*. Jun 2024;61(6):511-519. doi:10.1080/02770903.2023.2297365
